# The 9*H*-Fluoren Vinyl Ether Derivative SAM461 Inhibits Bacterial Luciferase Activity and Protects *Artemia franciscana* From Luminescent Vibriosis

**DOI:** 10.3389/fcimb.2018.00368

**Published:** 2018-11-08

**Authors:** Alberto J. Martín-Rodríguez, Sergio J. Álvarez-Méndez, Caroline Overå, Kartik Baruah, Tânia Margarida Lourenço, Parisa Norouzitallab, Peter Bossier, Víctor S. Martín, José J. Fernández

**Affiliations:** ^1^Instituto Universitario de Bio-Orgánica “Antonio González”, Centro de Investigaciones Biomédicas de Canarias, Universidad de La Laguna, Tenerife, Spain; ^2^Department of Microbiology, Tumor and Cell Biology, Karolinska Institutet, Stockholm, Sweden; ^3^Institute of Biophysics and Biophysical Chemistry, University of Regensburg, Regensburg, Germany; ^4^Laboratory of Aquaculture & Artemia Reference Center, Department of Animal Sciences and Aquatic Ecology, Faculty of Bioscience Engineering, Ghent University, Ghent, Belgium; ^5^Department of Animal Nutrition and Management, Faculty of Veterinary Medicine and Animal Sciences, Swedish University of Agricultural Sciences, Uppsala, Sweden; ^6^Laboratory of Immunology and Animal Biotechnology, Department of Animal Sciences and Aquatic Ecology, Ghent University, Ghent, Belgium

**Keywords:** vinyl ether, luciferase, *Artemia*, vibriosis, alternative anti-infectives

## Abstract

*Vibrio campbellii* is a major pathogen in aquaculture. It is a causative agent of the so-called “luminescent vibriosis,” a life-threatening condition caused by bioluminescent *Vibrio* spp. that often involves mass mortality of farmed shrimps. The emergence of multidrug resistant *Vibrio* strains raises a concern and poses a challenge for the treatment of this infection in the coming years. Inhibition of bacterial cell-to-cell communication or quorum sensing (QS) has been proposed as an alternative to antibiotic therapies. Aiming to identify novel QS disruptors, the 9H-fluroen-9yl vinyl ether derivative SAM461 was found to thwart *V. campbellii* bioluminescence, a QS-regulated phenotype. Phenotypic and gene expression analyses revealed, however, that the mode of action of SAM461 was unrelated to QS inhibition. Further evaluation with purified *Vibrio fischeri* and NanoLuc luciferases revealed enzymatic inhibition at micromolar concentrations. *In silico* analysis by molecular docking suggested binding of SAM461 in the active site cavities of both luciferase enzymes. Subsequent *in vivo* testing of SAM461 with gnotobiotic *Artemia franciscana* nauplii demonstrated naupliar protection against *V. campbellii* infection at low micromolar concentrations. Taken together, these findings suggest that suppression of luciferase activity could constitute a novel paradigm in the development of alternative anti-infective chemotherapies against luminescent vibriosis, and pave the ground for the chemical synthesis and biological characterization of derivatives with promising antimicrobial prospects.

## Introduction

Bacterial cell-to-cell communication or quorum sensing (QS) is a population-density-dependent extracellular signaling process that enables the coordination of collective behaviors in several bacterial species. This intercellular communication system relies on the synthesis, secretion and detection of signaling molecules termed autoinducers (AIs), which enable bacteria to optimize their metabolic resources and carry out tasks that are only possible at high cellular densities. Thus, QS exerts a tight control over bacterial gene expression, often involving hundreds of genes (Wilder et al., 2011; Majerczyk et al., 2016; Ball et al., 2017). Some of the QS-regulated physiological processes in diverse bacterial models include biofilm formation, host colonization and virulence factor production (Zhu and Mekalanos, 2003; Bassler and Losick, 2006; Waters et al., 2008; Ruwandeepika et al., 2011a; Bjelland et al., 2012). For this reason and given that, in principle, signal interference would not impose a selective pressure on bacterial populations, QS disruption has been proposed as a more selective target in the development of antibacterial therapies (LaSarre and Federle, 2013).

*Vibrio campbellii* is a marine bacterium whose bioluminescence is controlled by a complex QS regulatory system. Three hybrid sensor kinases, LuxN, LuxPQ, and CqsS responding to three different AIs converge in a response regulator, LuxO that controls the transcription of the mRNA encoding the master regulator of the QS regulon, LuxR. Upon DNA binding, LuxR enables the expression of hundreds of genes including the luciferase structural operon *luxCDABEGH*, where *luxAB* encode the two subunits of the bacterial luciferase (Meighen, 1991; Waters and Bassler, 2006). LuxG and LuxH are not essential for light production, though, and are not present in other bacterial *lux* homologs (Waidmann et al., 2011). Because of its QS-regulated light production and its well-characterized QS system, *V. campbellii* has been widely employed as a model biosensor to screen for QS inhibitors (Martín-Rodríguez and Fernández, 2016).

*V*. *campbellii* is a pernicious pathogen in aquaculture, affecting farm stocks of fish, shrimps and mollusks worldwide (Austin and Zhang, 2006; Haldar et al., 2011; Wang et al., 2015). Diseases caused by *V. campbellii* include skin ulcers, vasculitis, gastrointestinal disorders and eye lesions in fish (Austin and Zhang, 2006; Shen et al., 2017) and the so-called “luminescent vibriosis” in crustaceans and mollusks, often involving mass mortality and extensive economic loss (Travers et al., 2009; Darshanee Ruwandeepika et al., 2012; Lio-Po, 2016). This disease owes its name to the bioluminescence displayed by its causative agents, primarily *V*. *campbellii* and *V*. *harveyi*. Mortality rates between 60 and 80% have been reported in abalone (*Haliotis tuberculata*), up to 85% in white shrimp (*Litopenaeus vannamei*) and up to 100% in salmonids (Defoirdt et al., 2007a), with global estimated costs for this disease exceeding $9 billion per year (Bondad-Reantaso et al., 2005). The indiscriminate use of antibiotics over decades has resulted in the emergence of multidrug-resistant *V. campbellii* strains (Scarano et al., 2014). The need for sustainable alternative therapies is even more urgent taking into account the tight regulations and growing public health concerns associated with the use of antibiotics in aquaculture (Defoirdt et al., 2011).

Experimental characterization of novel drug candidates for aquaculture requires representative and reliable animal models. In this context, the *Artemia franciscana* naupliar gnotobiotic model is well-established, with the nauplii being relatively easy to rear under germ-free conditions and providing the additional advantage of eliminating any indirect effects caused by host microbiota, thereby allowing a direct cause-effect association during drug candidate testing (Baruah et al., 2015). In an effort to find QS antagonists from chemical libraries, SAM461 was identified as a potent inhibitor of *V. campbellii* bioluminescence with no inhibitory effect on bacterial growth at effective doses in the low-micromolar range. Here we describe our characterization of its mode of action and *in vivo* performance using axenically-hatched *A. franciscana* nauplii.

## Materials and methods

### Strains and growth conditions

The *V. campbellii* strains used in this study are listed in Table 1. Bacteria were recovered from cryopreserved stocks on marine agar (Difco). Single colonies were used to start the experiments as described below. When necessary, ampicillin (100 μg ml^−1^) and isopropyl-β-D-thiogalactoside (IPTG; 200 μM) were supplemented.

**Table 1:** Strains and primers used in this study.

**Strains**	**Genotype and relevant characteristics**	**Source or references**
*Vibrio campbellii* ATCC-BAA 1116 (BB120)	Wild type strain.	Bassler et al., 1994
*Vibrio campbellii* JAF548 pAK*lux*1	Strain JAF548 (BB120 *luxO* D47E linked to Kan^r^) carrying plasmid pAK*lux*1 (Amp^r^), a pBBR1MCS-4 derivative containing the *luxCDABE* operon from *Photorhabdus luminescens*. Luminescence independent of quorum sensing.	Defoirdt et al., 2012
**Primers**	**Sequence**	**Source or references**
qVhluxR_F	TCAATTGCAAAGAGACCTCG	Defoirdt et al., 2007b
qVhluxR_R	AGCAAACACTTCAAGAGCGA	Defoirdt et al., 2007b
qVHluxA_F	ATTTGCCGCAACTTCTTGGG	This study
qVHluxA_R	TGGTGTCTTTGTGGCCTTTC	This study
qVHluxC_F	AGATGCATTCGCCGCAAAAG	This study
qVHluxC_R	AACGTTGAAGTGGTCGCATG	This study
qVhrpoA_F	CGTAGCTGAAGGCAAAGATGA	Defoirdt et al., 2007b
qVhrpoA_R	AAGCTGGAACATAACCACGA	Defoirdt et al., 2007b

### Synthesis of SAM461

(*E*)-Methyl 3-((9*H*-fluoren-9-yl)oxy)acrylate (SAM461) was synthesized using 9-hydroxyfluorene (380 mg, 2.00 mmol) as starting material. Methyl propiolate (1.3 equiv) was added portionwise under Ar atmosphere (six portions, one portion every 5 min) to a solution of the alcohol (1 equiv) and DABCO (0.1 equiv) in dry dichloromethane (0.4 M). After thin-layer chromatography (TLC) analysis revealed a complete reaction (1 h approximately), the product was concentrated and purified by flash chromatography (28 cm of height of silica gel, *n*-hexane/Et_2_O 85/15) affording SAM461 (515 mg, 97%) as a yellowish solid consistent with reported data (Tejedor et al., 2014). SAM461 molecular weight (MW) and octanol:water partition coefficient (cLogP) were calculated with ChemBioDraw Ultra 13.0.0.3015 (CambridgeSoft, PerkinElmer).

Spectroscopic data of SAM461: *R*_F_ 0.38 (*n*-hexane/Et_2_O 80/20 two times); ^1^H-NMR (400 MHz, δ, CDCl_3_) 3.64 (s, 3H), 5.47 (d, *J* = 12.3 Hz, 1H), 5.94 (s, 1H), 7.28–7.35 (m, 2H), 7.40–7.47 (m, 3H), 7.55 (d, *J* = 7.5 Hz, 2H), 7.67 (d, *J* = 7.5 Hz, 2H); ^13^C-NMR (100 MHz, δ, CDCl_3_) 51.1 (q), 82.7 (d), 99.4 (d), 120.3 (d, 2C), 125.5 (d, 2C), 128.1 (d, 2C), 130.0 (d, 2C), 140.9 (s, 2C), 141.1 (s, 2 C), 160.8 (s), 168.0 (s); MS (EI) m/z (relative intensity) 266 (M)^+^ (1), 166 (30), 165 (100), 163 (11), 139 (5), 115 (3); HRMS calcd for C_17_H_14_O_3_ (M)^+^ 266.0943, found 266.0942. ^1^H and ^13^C-NMR spectra (Figure S1) were recorded on Bruker Avance instruments at room temperature, and data were processed using Topspin software (version 2.1); chemical shifts (δ) are reported in parts per million (ppm), and coupling constants (*J*) are quoted in Hertz (Hz); ^1^H-NMR spectrum is referenced to the resonance from residual CHCl_3_ at 7.250 ppm and multiplicity is expressed by the abbreviations m (multiplet), s (singlet) and d (doublet); ^13^C-NMR spectrum is referenced to the central peak of the signal from CDCl_3_ at 77.00 ppm, multiplicity was assigned from DEPT135 and DEPT90 experiments and is expressed by the abbreviations s (C), d (CH) and q (CH_3_). Mass spectra were recorded with an AutoSpec Micromass spectrometer by using electronic impact (EI-TOF 70 eV).

### Growth curves and quorum sensing assays with *vibrio campbellii*

Quorum sensing inhibition assays were performed in autoinducer bioassay (AB) medium (17.5 g l^−1^ NaCl, 12.3 g l^−1^ MgSO_4_, 2.0 g l^−1^ casamino acids, 0.01 M potassium phosphate, 0.001 M L-arginine, 1% v/v glycerol) as previously described (Martín-Rodríguez and Fernández, 2016). Briefly, diluted overnight cultures (1:100) were exposed to serial dilutions of SAM461 in sealed, white, clear bottom 96-well plates (Costar 3610). To keep solvent concentration to a minimum, a highly concentrated stock solution of SAM461 in DMSO was used (80 mM). Control experiments involved non-treated cells (untreated control) and cells supplemented with a volume of DMSO equivalent to that of the highest treatment dose (solvent control). Luminescence and optical density at 600 nm were measured every 15 min for 18 h in a multimode plate reader (PerkinElmer EnSpire). Luminescence reads of treatments were normalized with respect to that of the controls, and dose-response curves were adjusted using a four-parameter non-linear regression model as implemented in GraphPad Prism v5 (Prism Software). Experiments were run in triplicate.

### RNA extraction, cDNA synthesis and qRT-PCR

Overnight *V. campbellii* BB120 cultures were diluted 1:100 in AB medium with (8 μM) and without SAM461. The untreated control received a proportional amount of DMSO (0.01% v/v). Three biological replicates were prepared per condition. Bacterial cultures were incubated aerobically at 30°C for 8 h before RNA was isolated with the High Pure RNA Isolation Kit (Roche) as recommended by the manufacturer. Residual genomic DNA was removed after treatment with 5U RNAse-free DNAse (Promega). Complementary DNA (cDNA) synthesis was performed with the First Transcriptor cDNA synthesis kit (Roche) according to the manufacturer's instructions using 1 μg of total RNA. Expression of *luxR, luxA* and *luxC* and *rpoD* was determined using the primers listed in Table 1. Quantitative PCR reactions were prepared with the SensiMix SYBR & Fluorescein Kit (Bioline) in sealed optical 96-well plates using a Bio-Rad MyIQ instrument. Gene expression for treated and untreated cells was calculated with the qbase+ software (Biogazelle) and the statistical significance of the differences was analyzed by a two-tailed Student's *t*-test. Significance was set at *P* = 0.05.

### Enzymatic assays with *vibrio fischeri* and nanoluc luciferases

Bacterial luciferase assays were conducted with commercial *Vibrio fischeri* luciferase (V_f_Luc) (Sigma-Aldrich L8507), which is a close analog of that of *V. campbellii*, as described previously (Cruz et al., 2011). Briefly, 2 μl of substrate and cofactor solution (final concentrations after addition of enzyme solution: 0.06% BSA, 0.64 mM decanal, 25 μM FMN, 0.5 mM NADH) were dispensed inside the wells of a 1,536-well white/solid bottom high base plate (Greiner 789175), to which either 25 or 50 nl of compound stock solution were added from a 384-well acoustically compatible compound plate (Greiner 788876) using an ATS-100 acoustic dispenser (EDC Biosystems; 250 μM-122 nM, 12 point-titration with duplicates, DMSO, tartrazine and pifithrin-α as controls). The mixture was incubated for 5 min and then 2 μl of enzyme solution were added to each well of the 1,536-well plate (final concentrations: 1.88 g ml^−1^ bacterial luciferase and FMN reductases, approximate protein concentration of 0.75 mg ml^−1^); enzyme buffer (100 mM pH 7.0 sodium phosphate buffer) was used as control. After a 3-min incubation period at room temperature in the dark, luminescence was monitored for 180 s using a ViewLux system (PerkinElmer) with the following settings: gain = high (23X); speed = high (0.5 μs); binning = 6X, flatfield corrected using NanoLuc (Promega) standard.

NanoLuc (NLuc) luciferase inhibition testing was performed as described previously (Dranchak et al., 2013). Thus, 2 μl of NLuc assay substrate (Nano-Glo luminescence assay, Promega) (final concentrations: 300 mM sodium ascorbate, 5 mM sodium chloride, 0.1% triton X-100, 20 μM coelenterazine in 1X PBS, pH 7.4) were dispensed into white solid-bottom 1,536 well plates (Greiner Bio One) with a BioRAPTR FRD (Beckman Coulter). Compounds were transferred to the plates in 25–50 nl by an Echo acoustic dispenser (Labcyte) in the concentration range of 244 nM to 250 μM along with DMSO and titrations of cilnidipine positive control from top concentration of 125 μM. NLuc substrate reagent and compounds were incubated for 10 min at room temperature and one volume secreted NLuc medium was added with a BioRAPTR FRD. NLuc enzyme luminescence was measured using a ViewLux plate reader (PerkinElmer).

### Docking of SAM461 to *vibrio campbellii* luciferase and nanoluc

Molecular docking was used to investigate the binding sites of SAM461 to both the *V. campbellii* luciferase alpha chain (V_c_Luc) and NLuc, whose crystal structures are available from the Protein Data Bank (PDB). The potential binding areas (cavities) were found using CavityPlus (Xu et al., 2018), which detects cavities in the structure and informs about potential allosteric sites based on motion correlation analyses. Structure coordinates for V_c_Luc (PDB ID: 3FGC; Laskowski and Swindells, 2011) and NLuc (PDB ID: 5IBO) were used, after the heteroatoms were removed prior to docking. The 3D models of the ligands were created using ChemDraw Professional (CambridgeSoft, PerkinElmer). Docking experiments were performed using VINA (Trott and Olson, 2010) via YASARA (Krieger and Vriend, 2014), and the runs were clustered according to a root-mean-square-deviation (RMSD) cut-off of 5 Å. A grid box was placed around the residues forming the cavity of interest, localizing the docking area. Interactions between protein and ligands were initially analyzed using LigPlot^+^ (Laskowski and Swindells, 2011), and the ligand-protein complex was further examined and imaged with UCSF Chimera (Pettersen et al., 2004).

### Hatching of axenic *artemia franciscana* nauplii

Approximately 60 mg of *A. franciscana* cysts originating from the Great Salt Lake, Utah, USA (EG Type, batch 21452, INVE Aquaculture) were hydrated in 9 ml of sterile artificial seawater for 1 h. Sterile seawater was prepared by adding 3.5% of Instant Ocean® synthetic sea salt (Aquarium Systems) to 1 l of distilled water and filter-sterilizing. The cysts were sterilized and decapsulated by treatment with 330 μl NaOH (32%) and 5 ml NaOCl (50%) under constant, 0.2-μm filtered aeration. The reaction was stopped after 2 min by addition of 5 ml Na_2_S_2_O_3_ (1%) and aeration was discontinued. The decapsulated cysts were washed, re-suspended in sterile seawater and incubated for 28 h under constant illumination (27 μE m^−2^s^−1^). The sterility of the hatched *A. franciscana* nauplii was verified by adding hatching water (500 μl) to a tube containing marine broth (Difco) as well as spread plating (100 μl) on marine agar (Difco), followed by incubation at 28°C for 5 days (Baruah et al., 2011). Experiments started with non-sterile nauplii were discarded.

### *Artemia franciscana* challenge assays and lethality tests

A survival dose-response relationship for SAM461 was determined as described previously (Baruah et al., 2015). Briefly, a group of 20 germ-free nauplii at developmental stage II (in which their mouth is open to ingest food particles) was transferred to sterile 40 ml glass tubes containing 10 ml of sterile artificial seawater. Working 1 mM solutions of SAM461 were prepared in sterile seawater (10 ml) from a stock solution of the compounds in DMSO. The DMSO concentration in the different experimental groups was adjusted as per the solvent concentration in the highest dose group. Treatments were supplemented with SAM461 (0.125–8 μM) and challenged with *V. campbellii* at 10^7^ cells ml^−1^. *A. franciscana* survival was scored after 2 days by counting the number of live nauplii. As controls, the following groups were maintained: untreated nauplii that were not challenged with *V. campbellii* (negative control), untreated nauplii that were challenged with *V. campbellii* (positive control), and nauplii treated with DMSO and challenged with *V. campbellii* (DMSO control). Each experiment was performed in five replicates. Prior to challenge assays, the cytotoxic effect of SAM461 (2–32 μM) was determined in germ-free *A. franciscana* nauplii in the absence of *V. campbellii*, otherwise as described above. Survival data were subjected to one-way analysis of variances (ANOVA) followed by Dunnett's *post-hoc* analysis as implemented in GraphPad Prism v5 (Prism Software, La Jolla, CA). Statistical significance was set at *P* = 0.05.

## Results

### SAM461 inhibits bacterial luminescence independently of quorum sensing

**Figure 1:**
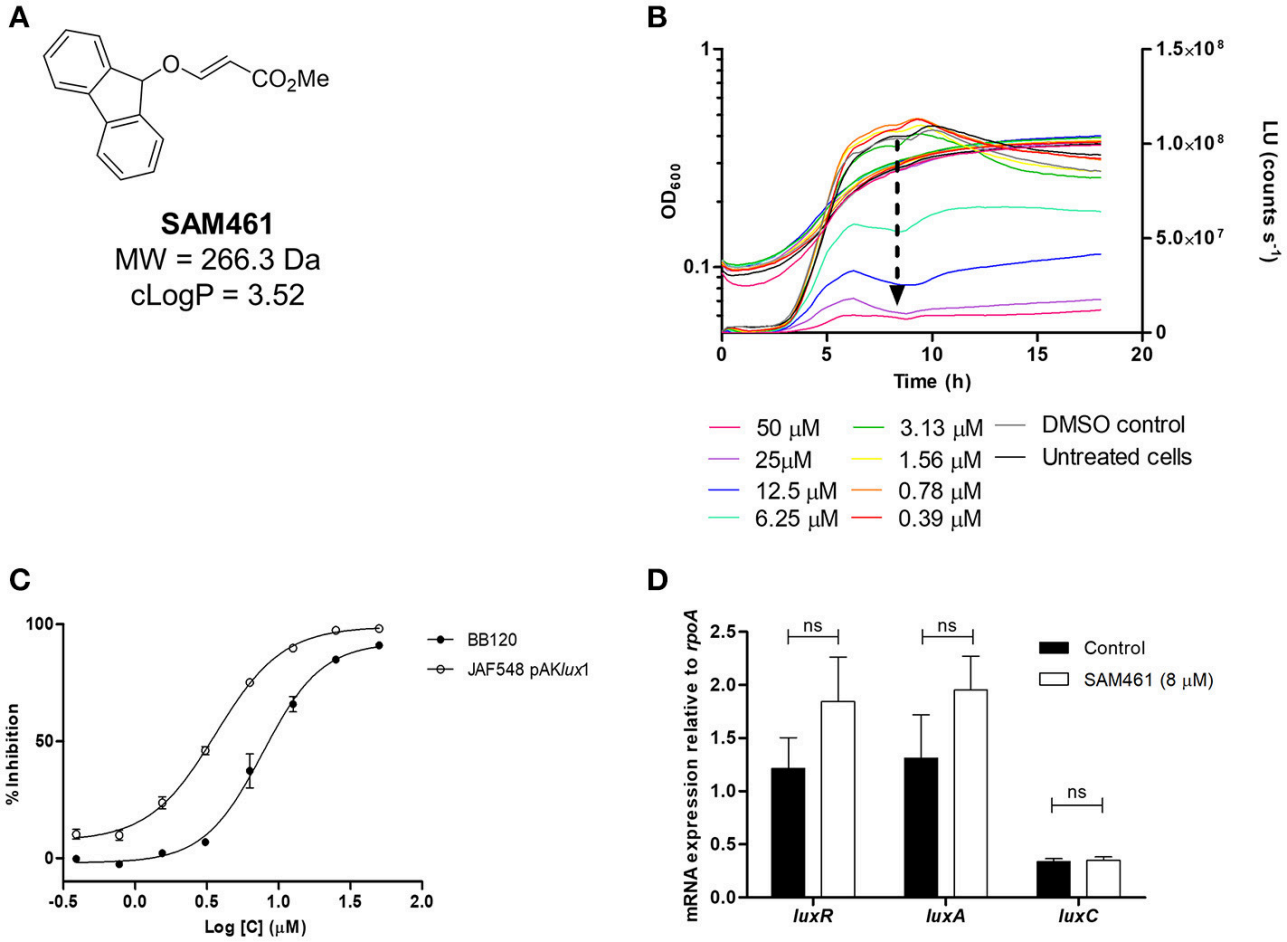
SAM461 inhibits *Vibrio campbellii* bioluminescence independently of quorum sensing. **(A)** Chemical structure and relevant chemical properties of SAM461. **(B)** *V. campbellii* BB120 growth (left *y* axis) and luminescence curves (right *y* axis) in the presence of SAM461 (0.39–50 μM), including solvent (DMSO) and untreated controls. The arrow highlights the dose-dependent luminescence quenching induced by SAM461. **(C)** Dose-response curves for SAM461-induced luminescence inhibition in *V. campbellii* BB120 (WT strain) and JAF548 pAK*lux*1 (constitutively luminescent mutant independent of QS). **(D)** Relative expression of *luxR, luxA*, and *luxC* in the absence (control) and presence (8 μM) of SAM461.

During the screening of diversity-oriented chemical libraries, compound SAM461 (Figure 1A) was identified as a bioluminescence inhibitor using *V. campbellii* BB120 as a bioreporter. SAM461 is a drug-like molecule fulfilling Lipinski's rule of 5 (Lipinski et al., 2001), a commonly used “rule of thumb” to determine the *druglikeliness* of a molecule. Therefore, SAM461 was synthesized in larger amounts to investigate its mode of action. Hence, we analyzed its effect on *V. campbellii* BB120 growth and bioluminescence in the concentration range 0.39–200 μM. SAM461 was found to be toxic to *V. campbellii* BB120 at concentrations >100 μM (data not shown), therefore these higher concentrations were excluded from further analysis. Testing of serial 2-fold dilutions of SAM461 from 50 to 0.39 μM revealed dose-dependent luminescence quenching (Figure 1B). The observed luminescence inhibition was not associated to alteration of bacterial growth rates at these doses, with only a slight growth delay being observed at the highest concentration of 50 μM (Figure 1B). To determine the potency of SAM461, dose-response curves were prepared. The IC_50_ for luminescence inhibition in *V. campbellii* BB120 was found to be 7.8 μM (Figure 1C). Taken together, these results indicate that SAM461 inhibits bacterial bioluminescence at non-toxic concentrations in the low micromolar range.

We initially hypothesized that SAM461 could be a QS inhibitor. Therefore, we performed the same experiment described above in a *V. campbellii* mutant displaying bioluminescence independently of QS. Thus, *V. campbellii* JAF548 is a BB120 isogenic mutant harboring a point mutation in the *luxO* allele that renders the cell constitutively non-luminescent (Defoirdt et al., 2012). Bioluminescence had been restored in this strain upon introduction of plasmid pAK*lux*1 harboring the *luxCDABE* operon from *Photorhabdus luminescens* under the *lac* promoter (Table 1). Hence, luminescence inhibition in this strain would indicate targets outside the QS circuit. SAM461 inhibited light production in this reporter strain similarly as in *V. campbellii* BB120 (IC_50_ = 3.6 μM, Figure 1C), thereby indicating the existence of targets beyond cell-to-cell communication.

To confirm that QS inhibition does not contribute to SAM461-induced luminescence quenching we determined the transcript levels of *luxR*, encoding the QS master regulator, as well as *luxC* and *luxA*, two of the QS-regulated genes in the *luxCDABEGH* operon, in treated (8 μM) and untreated *V. campbellii* BB120 cultures. The expression of these three genes was found to be not significantly different in treated and untreated *V. campbellii* cells (Figure 1D). This confirms unambiguously that SAM461 activity is independent of QS disruption.

### SAM461 inhibits luciferase activity

**Figure 2:**
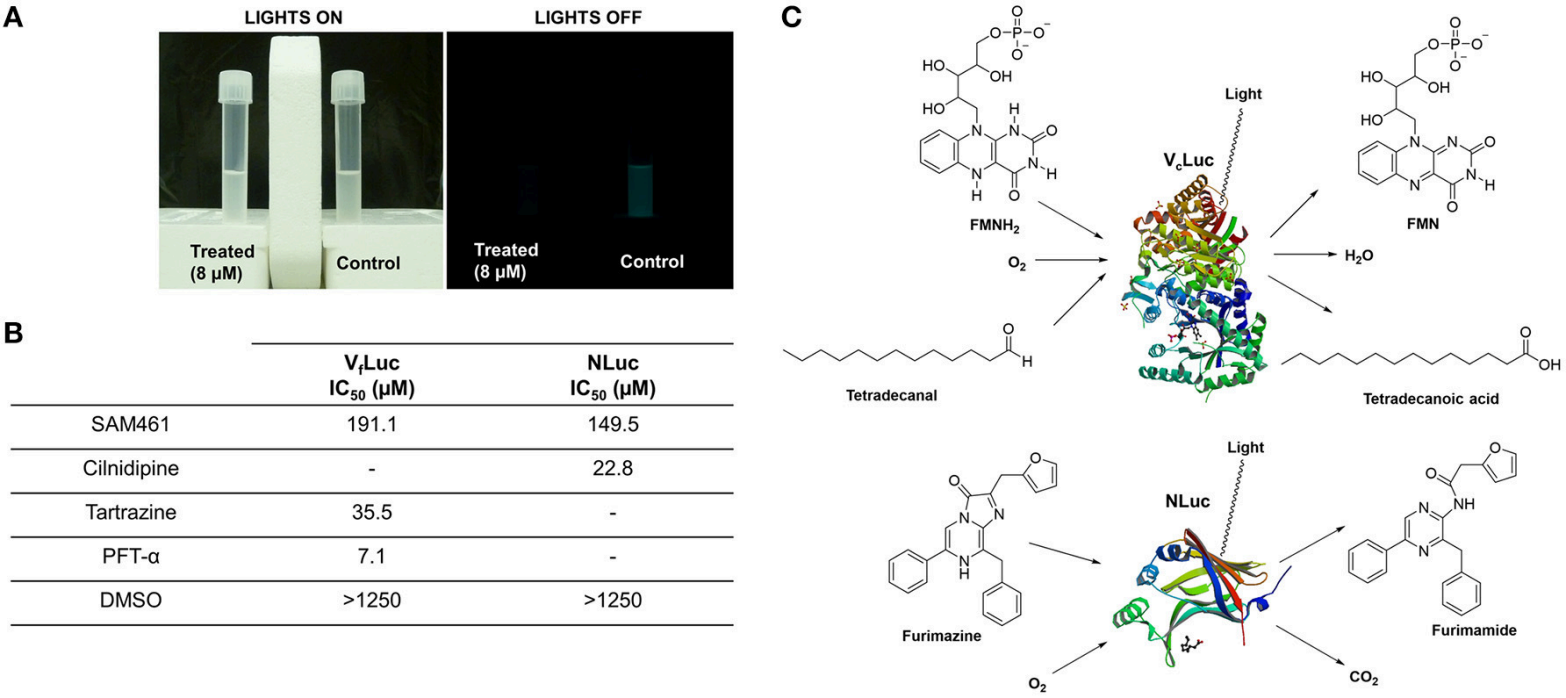
SAM461 interferes with bacterial luciferase activity. **(A)** Phenotypic evidence of potent bioluminescence inhibition caused by SAM461 (8 μM) in *Vibrio campbellii* BB120. **(B)** Half-maximal inhibitory concentration of SAM461 for *Vibrio fischeri* luciferase (V_f_Luc) and NanoLuc luciferase (NLuc). Tartrazine and PFT-α were used as positive controls for V_f_Luc inhibition, and cilnidipine was used as positive control for NLuc inhibition. DMSO was used as solvent control. **(C)** Substrates, cofactors and products involved in the redox processes catalyzed by the bacterial luciferase (*V. campbellii*, V_c_Luc) and NLuc resulting in light emission. Protein structures were retrieved from the Protein Data Bank (PDB: 3FGC–V_c_Luc, and 5IBO–NLuc).

We have shown that SAM461 displays potent bioluminescence inhibition in *V*. *campbellii* at low micromolar doses in a QS-independent fashion. We therefore reasoned that the potent effect observed in live bacteria (Figure 2A) could be due to inhibition of the bacterial luciferase enzyme. Using purified *V. fischeri* luciferase (V_f_Luc), we measured enzyme activity in the presence of serial dilutions of SAM461. Indeed, SAM461 inhibited V_f_Luc *in vitro* with an IC_50_ = 191.1 μM, indicating a moderately potent activity in comparison to other V_f_Luc inhibitors, such as tatrazine and PFT-α used as controls (Kim and Spiegel, 2013; Figure 2B). To determine the specificity of SAM461, we further tested this compound against NLuc, which is structurally and biochemically different to the bacterial luciferase (Figure 2C). SAM461 was found to inhibit NLuc activity with an IC_50_ = 149.5 μM, a similar value to that determined for V_f_Luc. Taken together, these findings suggest that SAM461 inhibits luciferase activity non-selectively.

### Molecular docking

To gain an insight on the molecular interactions of SAM461 and its luciferase protein targets, an *in silico* analysis by molecular docking was performed with the crystal structures of *V. campbellii* luciferase (V_c_Luc) and NLuc.

#### Analysis of SAM461-V_c_Luc interactions

**Figure 3:**
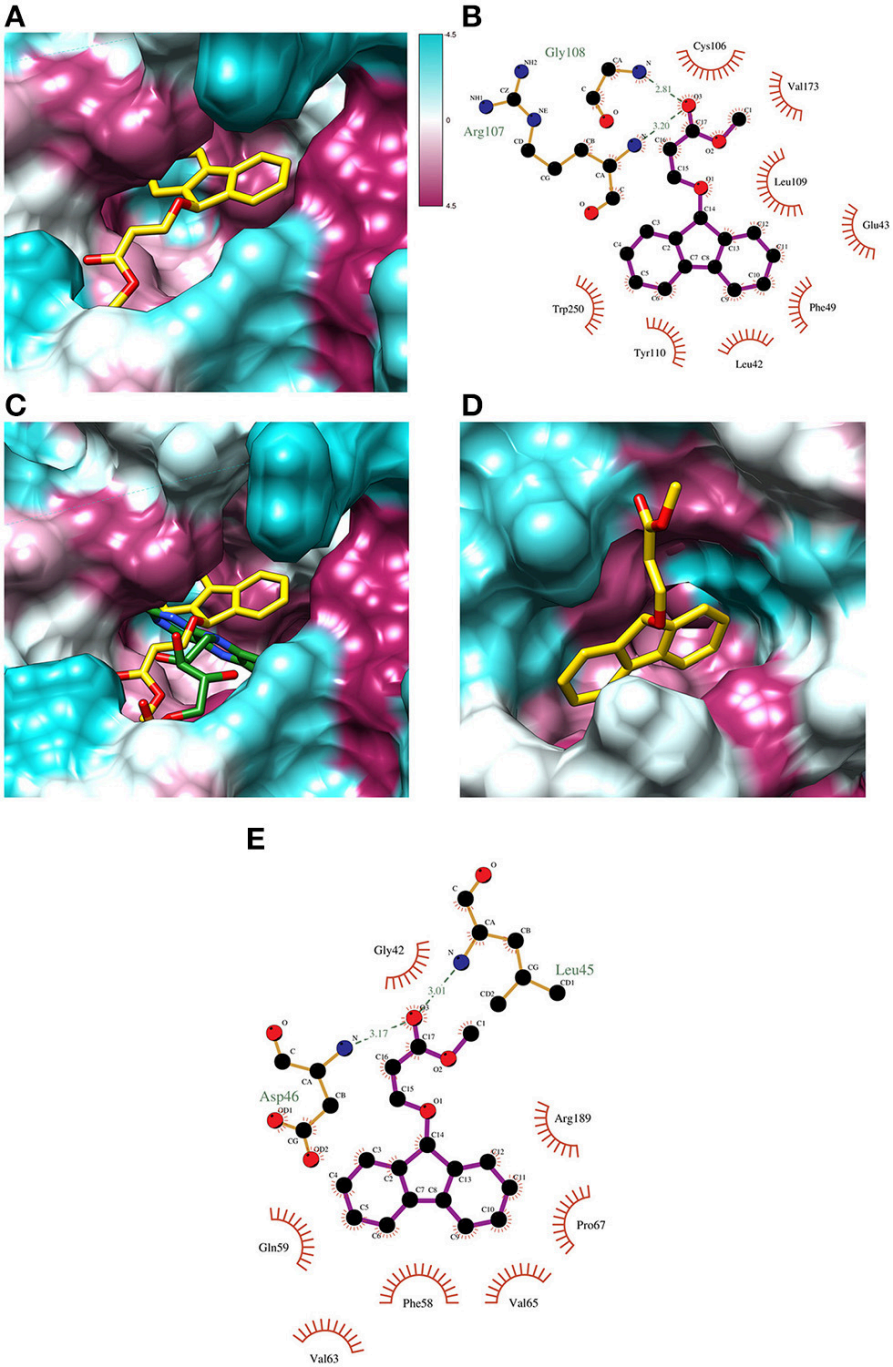
Docking poses of ligand/substrate and their calculated interactions. **(A)** Docking pose of SAM461 (second highest scoring) in the predicted active site pocket of V_c_Luc. The images show the hydrophobic surface areas marked as magenta, and hydrophilic areas in cyan based on the hydrophobicity scale of Kyte and Doolittle (57). **(B)** Interaction diagram of the docked SAM461 molecule in the V_c_Luc active site. The interactions represent the second docking cluster, displaying hydrogen bonds together with hydrophobic contacts. The dotted green lines represent hydrogen bonds. Red lines indicate hydrophobic interactions between protein residues and ligand atoms. **(C)** Comparison of the X-ray structure of the FMNH-V_c_Luc complex (green; PDB: 3FGC) and the docking pose of SAM461 (yellow) in the active site pocket of V_c_Luc. The general poses of the molecules are similar, with the rings turned toward the cavity interior and the flexible carbon chains forming hydrogen bonds with residues closer to the cavity opening. **(D)** The docked SAM461 ligand in the active site cavity of NLuc. **(E)** Interaction diagram of the SAM461 ligand docked to the active site cavity of NLuc. The hydrogen bonds (shown as dotted green lines) likely act to stabilize the flexible region of the molecule.

Putative allosteric sites in V_c_Luc were detected by CavityPlus based on the cavity containing the active site. Since the X-ray structure of V_c_Luc was solved in complex with the substrate FMNH_2_, docking of FMNH_2_ was used to test the reliability of the docking results. For both SAM461 and FMNH, 400 docking poses were generated in the putative allosteric sites and the active site. To evaluate the docking results of SAM461 and FMNH_2_, the binding energy (kcal mol^−1^) and calculated affinity (CA; μM) of the docked ligands were considered. Docking of SAM461 to the different cavities of V_c_Luc revealed a significantly different binding energy and CA (over 15-fold) when docked in the FMNH_2_ binding site compared to the potential allosteric cavities (Table 2). The highest scoring pose in the first cluster exhibited a binding energy of −8.7 kcal mol^−1^ and a CA of 0.36 μM. However, the highest scoring pose in the second cluster is hydrogen bonded, establishing an interaction of SAM461 with Arg107 and Gly108 (Figures 3A,B). Due to the apparent flexibility of the acrylate chain, the hydrogen bond interactions would likely stabilize this binding pose in the cavity.

**Table 2:** Docking binding energy and calculated affinity of the highest scores in the different cavities of *Vibrio campbelli* luciferase (V_c_Luc).

**Ligand**	**Cavity V_c_Luc**	**Binding energy (kcal mol^−1^)**	**Calculated affinity (μM)**
**SAM461**	FMNH_2_ binding site (hydrogen bond)	−7.6	2.3
	FMNH_2_ binding site (highest score)	−8.7	0.36
	Potential Allosteric site 1	−6.5	14.8
	Potential Allosteric site 2	−7.0	6.6
	Potential Allosteric site 3	−7.0	6.8
**FMNH**_2_	FMNH_2_ binding site	−9.1	0.21
**Cavity NLuc**			
**SAM461**	Potential Active Site	−6.7	11.9
	Potential Allosteric site	−5.9	41.4
**Furimazine**	Potential Active site	−7.4	3.8
	Potential Allosteric site	−7.0	7.1

*Potential allosteric sites were detected by CavityPlus*.

Docking of FMNH_2_ to V_c_Luc yielded a similar pose as that observed in the X-ray structure (Figure S2A). Because the aliphatic chain in this molecule is also likely to be flexible, differences in the chain orientation were observed. Despite this apparent flexibility, both the docked and crystal resolved molecule (Laskowski and Swindells, 2011) formed very similar hydrogen bonds and residue contacts. They shared hydrogen bonds with Glu43, Ala75, Arg107, Leu109, Glu175, Ser176 and Thr179, and multiple shared hydrophobic interactions with other residues (Figures S2B,C). The hydrogen bond to Arg107 was also observed for SAM461. Docking of FMNH_2_ displayed a calculated binding energy of −9.1 kcal mol^−1^ and a CA of 0.21 μM. Superposing the ligand-receptor complexes showed a similar orientation of both SAM461 and FMNH_2_, with the rigid aromatic rings against the hydrophobic cavity, and the flexible region pointing toward the cavity opening (Figure 3C).

#### Analysis of SAM461-NLuc interactions

NLuc does not have a confirmed substrate binding site, but it is assumed that the active site is located in the central cavity, since it should be able to accommodate the substrate coelenterazine (Laskowski and Swindells, 2011). CavityPlus detected one possible allosteric cavity based on this active site. Furimazine was docked to both the active and potential allosteric site to compare with the results of SAM461. In contrast to V_c_Luc, the docked binding energy and CA for NLuc were not as different between the two cavities, but the active site provided a higher calculated affinity for both ligands (Table 2). The highest scoring SAM461 pose in the active site has the fluorene rings oriented toward the hydrophobic interior of the cavity, and the acrylate chain turning outwards (Figure 3D). This pose allows hydrogen bonding with Leu45 and Asp46, and hydrophobic contacts with 7 other residues (Figure 3E). The highest scoring pose of furimazine in the same site did not form hydrogen bonds, but 11 hydrophobic contacts, many of which are shared with the SAM461 pose (Figure S2D). When investigating the areas surrounding the ligand, the larger cavity housing the active site may provide better solvent protection for both furimazine and SAM461 (Figures S2E,F).

### SAM461 protects *artemia franciscana* from *vibrio campbellii* infection

**Figure 4:**
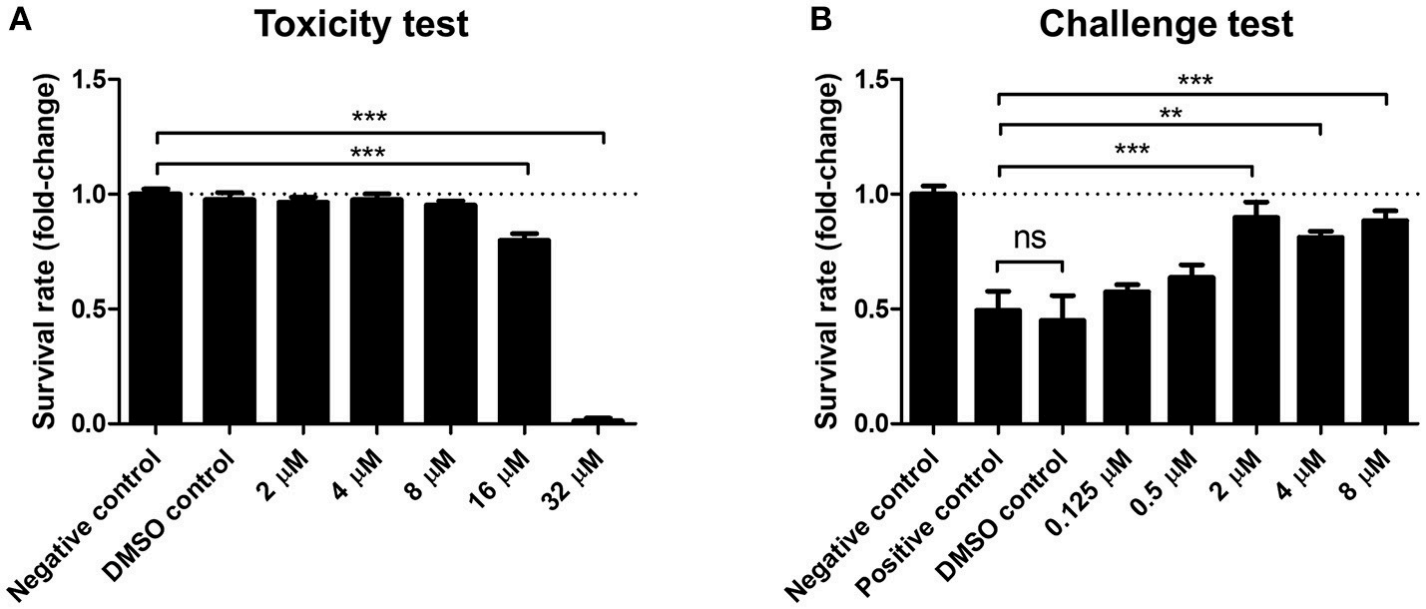
SAM461 confers protection to *Artemia franciscana* nauplii against *Vibrio campbellii* BB120 at non-toxic concentrations. **(A)** Toxicity of SAM461 to germ-free *A. franciscana*. Nauplii were exposed to SAM461 at the indicated doses. Un-exposed nauplii served as negative control. Nauplii exposed to only DMSO served as solvent control. Survival was scored after 48 h of exposure. Data are presented as fold-change relative to the negative control, which has been normalized to 1 (dotted line). Data represent the mean ± standard error of five replicates. Asterisks indicate significant differences relative to the negative control (^***^P < 0.001). **(B)** Survival rate of *A. franciscana* nauplii during co-challenge with *V*. *campbellii*. SAM461 was added to the culture water at indicated concentrations. Simultaneously, the nauplii were challenged with *V. campbellii* at 10^7^ cfu ml^−1^ for 48 h. Control groups included untreated nauplii challenged with *V. campbellii* (positive control), DMSO-treated nauplii challenged with *V. campbellii* (DMSO control) and non-challenged (uninfected) nauplii (negative control). Data are presented as fold-change relative to the negative control, which has been normalized to 1 (dotted line). Values represent the mean ± standard error of five replicates. Asterisks indicate significant differences with respect to the positive control (^**^P < 0.01; ^***^P < 0.001).

Light production is a major metabolic endeavor, and dark mutants of pathogenic *Vibrio* are known to be less virulent than their wild-type counterparts (Phuoc et al., 2009; Ruwandeepika et al., 2011b). To determine the effect of SAM461 on *V. campbellii* infectivity we used the gnotobiotic *A*. *franciscana* infection model (Baruah et al., 2015). We first determined the toxicity of SAM461 toward *A. franciscana* nauplii in the range 2–32 μM. The lowest dose of the compound exerting significant toxicity was 16 μM, whereas no toxicity was detected in the range 2–8 μM (Figure 4A). We next challenged germ-free *A*. *franciscana* nauplii with *V*. *campbellii* in the absence and presence of SAM461 at non-toxic doses (0.125–8 μM). SAM461 fully protected *A. franciscana* from *V. campbellii* infection at concentrations as low as 2 μM (*P* < 0.001, Figure 4B). At this dose, *A. franciscana* survival was increased 2-fold in comparison to untreated nauplii (Figure 4B), thereby highlighting the therapeutic potential of this molecule.

## Discussion

Luminescent vibriosis caused by *V. campbellii* and close relatives is a major disease with a remarkable economic impact. Growing concerns related to the use of antibiotics in aquaculture and the emergence of multidrug resistant bacterial pathogens have motivated a global search of alternative therapeutic and prophylactic options (Defoirdt et al., 2007a, 2011). In this context, QS inhibitors have been proposed as promising candidates (Bhardwaj et al., 2013; LaSarre and Federle, 2013; Kim et al., 2018). Searching for potential QS inhibitors from in-house chemical libraries (University of La Laguna, Spain), compound SAM461 was identified as a hit during an initial screening round involving testing of the chemicals against *V. campbellii* BB120.

In this study we have delineated the mode of action of SAM461. Given the known association between light production and QS in *V. campbellii*, we initially envisioned that the activity exhibited by SAM461 could be related to QS inhibition. However, testing of SAM461 against a genetically-engineered, constitutively luminescent *V. campbellii* mutant and subsequent transcriptional analysis of *luxR* and QS-regulated genes revealed a mode of action independent of QS disruption. This was not completely surprising, though. *V*. *campbellii* produces three types of QS signaling molecules: AI-1, an *N*-acyl homoserine lactone; AI-2, a furanosyl borate diester; and CAI-1, a long-chain amino ketone (Anetzberger et al., 2012). Even though examples of QS inhibitors without chemical relatedness to the natural autoinducers exist, most of the known QS disruptors are chemical analogs of the native signal ligands (Galloway et al., 2011; Kalia, 2013; Martín-Rodríguez et al., 2016), which is not the case of SAM461. Nevertheless, with a half-maximal inhibition value in the single-digit μM range and no toxicity in the bacterial population as determined by growth inhibition at effective doses, the activity of this molecule deserved further characterization.

We reasoned that the bioluminescence inhibition observed in *V*. *campbellii* JAF548 pAK*lux*1 could be due to impaired luciferase activity. Recall that in this dark mutant genetic background the *lux* operon is ectopically expressed, thus rendering the cells constitutively bright independently of the cell population density. Indeed, SAM461 inhibited bacterial luciferase activity *in vitro* with an IC_50_ of 191.1 μM. This IC_50_ was 2 orders of magnitude higher than that observed in live bacteria. These differences between *in vitro* and *in vivo* activities are not uncommon, and they have been reported for other bacterial luciferase inhibitors in *V. campbellii* as well (Kim and Spiegel, 2013).

The biochemistry behind light production in *Vibrio* spp. is complex (Kim and Spiegel, 2013). The bacterial luciferase has three substrates: reduced flavin mononucleotide (FMNH_2_), a long-chain fatty acid aldehyde (usually tetradecanal) and molecular oxygen. FMNH_2_ is the product of the reduction of FMN by NADPH, a reaction catalyzed by the enzyme NADPH FMN oxidoreductase. FMNH_2_ is transferred to the bacterial luciferase, where it is oxidized by molecular oxygen. This results in the formation of hydroperoxide that reacts with a fatty acid aldehyde produced by the fatty acid reductase complex. The reaction of the aldehyde with hydroperoxide results in the generation of an excited-state intermediate that emits blue-green light and gives H_2_O and FMN as products. An overview of this complex process has been presented in Figure 2C. Hence, interference of SAM461 with the luciferase enzyme or any of the proteins or metabolic pathways involved in substrate and cofactor biosynthesis could lead to decreased light emission. The biochemistry of the NLuc luciferase is different. This enzyme is an engineered derivative of the naturally-occurring *Oplophorus gracilirostris* luciferase and exhibits high substrate specificity. Thus, NLuc catalyzes the oxidation of furimazine to produce furimamide, carbon dioxide and an intense light output. Testing of SAM461 against NLuc revealed similar performance as against the bacterial luciferase, and therefore we deduced that SAM461 is a non-selective luciferase inhibitor.

Although the docking experiments do not aim for the validation of whether the SAM461 ligand binds in the active site of its luciferase protein targets, our *in silico* results demonstrate the chemical feasibility of such a scenario. Docking of SAM461 and FMNH_2_ showed similar theoretical binding energies and calculated affinities when bound to the active site of V_c_Luc. While these values might differ from the actual thermodynamic parameters, the 15-fold decrease of CA determined for the active site with respect to the allosteric site strongly suggest that both ligands have a preferred theoretical binding toward the former. The observation that the FMNH_2_ substrate displays similar binding poses to the binding conformation of the flavin moiety in the X-ray protein-substrate complex, but with differences in the aliphatic chain conformation, supports the assumed flexibility. Determining the binding of NLuc was more demanding since the ligands showed similar affinity toward both the active and allosteric binding sites, with furimazine having stronger apparent affinity than SAM461 for both cavities. The larger cavity harboring the active site could provide a better binding pocket for both the native ligand furimazine and SAM461. The potential allosteric site would possibly not offer enough protection against the solvent. This together with the slightly preferred affinity toward the active site could suggest SAM461 binds to the active site of NLuc.

Luminescence has been reported to play a role in host colonization and infectivity in both commensal and pathogenic *Vibrio* spp. For example, luminescence genes have been shown to play an important role in the symbiotic colonization of the luminescent organs of the squid host by *V. fischeri* (Visick et al., 2000; Nyholm and McFall-Ngai, 2004; Chun et al., 2008). In the fish pathogen *V. salmonicida, luxA* mutants showed impaired infectivity and were outcompeted by the WT strain in co-challenge tests in Atlantic salmons (Nelson et al., 2007). In *V. campbellii* BB120 specifically, non-luminescent variants have been found to be less virulent than their luminescent counterparts in *A*. *franciscana* (Phuoc et al., 2009; Ruwandeepika et al., 2011b). Consistent with these precedents, the luminescence inhibitor SAM461 was found to protect *A. franciscana* from *V. campbellii* infection at low micromolar doses. Previous studies have found decreased virulence factor production and increased susceptibility to host defense mechanisms in non-luminescent variants of pathogenic *Vibrio* spp. (Szpilewska et al., 2003; Katsev et al., 2004; Phuoc et al., 2009; Ruwandeepika et al., 2011b), phenomena that could contribute to the observed performance of SAM461 during *in vivo* infection experiments.

In conclusion, we have presented herein that targeting the bacterial luciferase could constitute a novel paradigm in the treatment of luminescent vibriosis. SAM461 is a small, drug-like vinyl ether that supports diverse functionalities in its skeleton (Tejedor et al., 2013; Zhu and Kirsch, 2013) thus streamlining diverse-oriented synthesis and subsequent analyses of structure-activity relationships based on this lead. The lack of chronic toxicity of SAM461 at effective doses on the bacterial pathogen as well as in the host results promising to prevent the emergence of bacterial resistance and encourages its potential use as an adjuvant chemotherapy.

## Ethics statement

This study is exempt from ethics committe approval since it only involves experimental research with invertebrate larvae, which is not subjected to animal research regulations.

## Author contributions

AJM-R conceived the idea of the work. AJM-R performed the *in vitro* experiments with *V. campbellii*. SJÁ-M synthesized compound SAM461. CO conducted the molecular docking analyses. KB, TL, PN, and PB designed the *in vivo* infection studies with gnotobiotic *A. franciscana* nauplii. TL and PN performed the challenge and toxicity tests. AJM-R wrote the manuscript with the input of all co-authors. All of the authors contributed to data analysis. VSM and JJF led the projects funding the study.

### Conflict of interest statement

The authors declare that the research was conducted in the absence of any commercial or financial relationships that could be construed as a potential conflict of interest.

## References

[B1] Anetzberger C. Schell U. Jung K. (2012). Single cell analysis of *Vibrio harveyi* uncovers functional heterogeneity in response to quorum sensing signals. BMC Microbiol. 12, 209. 10.1186/1471-2180-12-209 22985329 PMC3511230

[B2] Austin B. Zhang X.-H. (2006). *Vibrio harveyi*: a significant pathogen of marine vertebrates and invertebrates. Lett. Appl. Microbiol. 43, 119–124. 10.1111/j.1472-765X.2006.01989.x 16869892

[B3] Ball A. S. Chaparian R. R. van Kessel J. C. (2017). Quorum sensing gene regulation by LuxR/HapR master regulators in vibrios. J. Bacteriol. 199, e00105–e00117. 10.1128/JB.00105-17 28484045 PMC5585708

[B4] Baruah K. Duy Phong H. P. P. Norouzitallab P. Defoirdt T. Bossier P. (2015). The gnotobiotic brine shrimp (*Artemia franciscana*) model system reveals that the phenolic compound pyrogallol protects against infection through its prooxidant activity. Free Radic. Biol. Med. 89, 593–601. 10.1016/j.freeradbiomed.2015.10.397 26459033

[B5] Baruah K. Ranjan J. Sorgeloos P. MacRae T. H. Bossier P. (2011). Priming the prophenoloxidase system of *Artemia franciscana* by heat shock proteins protects against *Vibrio campbellii* challenge. Fish Shellfish Immunol. 31, 134–141. 10.1016/j.fsi.2011.04.008 21554959

[B6] Bassler B. L. Losick R. (2006). Bacterially speaking. Cell 125, 237–246. 10.1016/j.cell.2006.04.001 16630813

[B7] Bassler B. L. Wright M. Silverman M. R. (1994). Sequence and function of LuxO, a negative regulator of luminescence in *Vibrio harveyi*. Mol. Microbiol. 12, 403–412. 10.1111/j.1365-2958.1994.tb01029.x 8065259

[B8] Bhardwaj A. K. Vinothkumar K. Rajpara N. (2013). Bacterial quorum sensing inhibitors: attractive alternatives for control of infectious pathogens showing multiple drug resistance. Recent Pat. Antiinfect. Drug Discov. 8, 68–83. 10.2174/1574891X11308010012 23394143

[B9] Bjelland A. M. Sørum H. Tegegne D. A. Winther-Larsen H. C. Willassen N. P. Hansen H. (2012). LitR of *Vibrio salmonicida* is a salinity-sensitive quorum-sensing regulator of phenotypes involved in host interactions and virulence. Infect. Immun. 80, 1681–1689. 10.1128/IAI.06038-11 22371373 PMC3347430

[B10] Bondad-Reantaso M. G. Subasinghe R. P. Arthur J. R. Ogawa K. Chinabut S. Adlard R.. (2005). Disease and health management in Asian aquaculture. Vet. Parasitol. 132, 249–272. 10.1016/j.vetpar.2005.07.005 16099592

[B11] Chun C. K. Troll J. V. Koroleva I. Brown B. Manzella L. Snir E.. (2008). Effects of colonization, luminescence, and autoinducer on host transcription during development of the squid-vibrio association. Proc. Natl. Acad. Sci. U.S.A. 105, 11323–11328. 10.1073/pnas.0802369105 18682555 PMC2516268

[B12] Cruz P. G. Auld D. S. Schultz P. J. Lovell S. Battaile K. P. MacArthur R.. (2011). Titration-based screening for evaluation of natural product extracts: identification of an aspulvinone family of luciferase inhibitors. Chem. Biol. 18, 1442–1452. 10.1016/j.chembiol.2011.08.011 22118678 PMC3225805

[B13] Darshanee Ruwandeepika H. A. Sanjeewa Prasad Jayaweera T. Paban Bhowmick P. Karunasagar I. Bossier P. Defoirdt T. (2012). Pathogenesis, virulence factors and virulence regulation of vibrios belonging to the Harveyi clade. Rev. Aquac. 4, 59–74. 10.1111/j.1753-5131.2012.01061.x

[B14] Defoirdt T. Benneche T. Brackman G. Coenye T. Sorgeloos P. Scheie A. A. (2012). A quorum sensing-disrupting brominated thiophenone with a promising therapeutic potential to treat luminescent vibriosis. PLoS ONE 7:e41788. 10.1371/journal.pone.0041788 22848604 PMC3404956

[B15] Defoirdt T. Boon N. Sorgeloos P. Verstraete W. Bossier P. (2007a). Alternatives to antibiotics to control bacterial infections: luminescent vibriosis in aquaculture as an example. Trends Biotechnol. 25, 472–479. 10.1016/j.tibtech.2007.08.001 17719667

[B16] Defoirdt T. Miyamoto C. M. Wood T. K. Meighen E. a Sorgeloos P. Verstraete W.. (2007b). The natural furanone (5Z)-4-bromo-5-(bromomethylene)-3-butyl-2(5H)-furanone disrupts quorum sensing-regulated gene expression in *Vibrio harveyi* by decreasing the DNA-binding activity of the transcriptional regulator protein LuxR. Environ. Microbiol. 9, 2486–2495. 10.1111/j.1462-2920.2007.01367.x 17803774

[B17] Defoirdt T. Sorgeloos P. Bossier P. (2011). Alternatives to antibiotics for the control of bacterial disease in aquaculture. Curr. Opin. Microbiol. 14, 251–258. 10.1016/j.mib.2011.03.004 21489864

[B18] Dranchak P. MacArthur R. Guha R. Zuercher W. J. Drewry D. H. Auld D. S.. (2013). Profile of the GSK published protein kinase inhibitor set across ATP-dependent and-independent luciferases: implications for reporter-gene assays. PLoS ONE 8:e57888. 10.1371/journal.pone.0057888 23505445 PMC3591448

[B19] Galloway W. R. Hodgkinson J. T. Bowden S. D. Welch M. Spring D. R. (2011). Quorum sensing in Gram-negative bacteria: small-molecule modulation of AHL and AI-2 quorum sensing pathways. Chem. Rev. 111, 28–67. 10.1021/cr100109t 21182299

[B20] Haldar S. Chatterjee S. Sugimoto N. Das S. Chowdhury N. Hinenoya A.. (2011). Identification of *Vibrio campbellii* isolated from diseased farm-shrimps from south India and establishment of its pathogenic potential in an *Artemia* model. Microbiology 157, 179–188. 10.1099/mic.0.041475-0 20847009

[B21] Kalia V. C. (2013). Quorum sensing inhibitors: an overview. Biotechnol. Adv. 31, 224–245. 10.1016/j.biotechadv.2012.10.004 23142623

[B22] Katsev A. M. Wegrzyn G. Szpilewska H. (2004). Effects of hydrogen peroxide on light emission by various strains of marine luminescent bacteria. J. Basic Microbiol. 44, 178–184. 10.1002/jobm.200310330 15162391

[B23] Kim B. S. Jang S. Y. Bang Y.-J. Hwang J. Koo Y. Jang K. K.. (2018). QStatin, a selective inhibitor of quorum sensing in *Vibrio* species. MBio 9, e02262–e02217. 10.1128/mBio.02262-17 29382732 PMC5790914

[B24] Kim T. Spiegel D. A. (2013). Serendipitous discovery of two highly selective inhibitors of bacterial luciferase. Tetrahedron 69, 7692–7698. 10.1016/j.tet.2013.05.086

[B25] Krieger E. Vriend G. (2014). YASARA view–molecular graphics for all devices–from smartphones to workstations. Bioinformatics 30, 2981–2982. 10.1093/bioinformatics/btu426 24996895 PMC4184264

[B26] LaSarre B. Federle M. J. (2013). Exploiting quorum sensing to confuse bacterial pathogens. Microbiol. Mol. Biol. Rev. 77, 73–111. 10.1128/MMBR.00046-12 23471618 PMC3591984

[B27] Laskowski R. A. Swindells M. B. (2011). LigPlot+: multiple ligand-protein interaction diagrams for drug discovery. J. Chem. Inf. Model. 51, 2778–2786. 10.1021/ci200227u 21919503

[B28] Lio-Po G. D. (2016). Luminous *Vibrio* and the greenwater culture of the tiger shrimp *Penaeus monodon* with *Tilapia*, in Tilapia in Intensive Co-culture, eds Perschbacher P. W. Stickney R. R. (Chichester: John Wiley & Sons, Ltd), 81–93.

[B29] Lipinski C. A. Lombardo F. Dominy B. W. Feeney P. J. (2001). Experimental and computational approaches to estimate solubility and permeability in drug discovery and development settings. Adv. Drug Deliv. Rev. 46, 3–26. 10.1016/S0169-409X(00)00129-0 11259830

[B30] Majerczyk C. Schneider E. Greenberg E. P. (2016). Quorum sensing control of type VI secretion factors restricts the proliferation of quorum-sensing mutants. Elife 5, e14712. 10.7554/eLife.14712 27183270 PMC4868534

[B31] Martín-Rodríguez A. Fernández J. (2016). A bioassay protocol for quorum sensing studies using *Vibrio campbellii*. Bio Protoc. 6:e1866 10.21769/BioProtoc.1866

[B32] Martín-Rodríguez A. Quezada H. Aragón G. de la Fuente-Nuñez C. Castillo-Juarez I. Maeda T. (2016). Recent advances in novel antibacterial development, in Frontiers in Clinical Drug Research: Anti-Infectives vol. 2, ed Atta-Ur-Rahman (Sharjah: Bentham Science Publishers), 3–61.

[B33] Meighen E. A. (1991). Molecular biology of bacterial bioluminescence. Microbiol. Rev. 55, 123–142. 2030669 10.1128/mr.55.1.123-142.1991 PMC372803

[B34] Nelson E. J. Tunsjø H. S. Fidopiastis P. M. Sørum H. Ruby E. G. (2007). A novel *lux* operon in the cryptically bioluminescent fish pathogen *Vibrio salmonicida* is associated with virulence. Appl. Environ. Microbiol. 73, 1825–1833. 10.1128/AEM.02255-06 17277225 PMC1828807

[B35] Nyholm S. V. McFall-Ngai M. J. (2004). The winnowing: establishing the squid-vibrio symbiosis. Nat. Rev. Microbiol. 2, 632–642. 10.1038/nrmicro957 15263898

[B36] Pettersen E. F. Goddard T. D. Huang C. C. Couch G. S. Greenblatt D. M. Meng E. C.. (2004). UCSF chimera–a visualization system for exploratory research and analysis. J. Comput. Chem. 25, 1605–1612. 10.1002/jcc.20084 15264254

[B37] Phuoc L. H. Defoirdt T. Sorgeloos P. Bossier P. (2009). Virulence of luminescent and non-luminescent isogenic vibrios towards gnotobiotic *Artemia franciscana* larvae and specific pathogen-free *Litopenaeus vannamei* shrimp. J. Appl. Microbiol. 106, 1388–1396. 10.1111/j.1365-2672.2008.04107.x 19187135

[B38] Ruwandeepika H. A. Bhowmick P. P. Karunasagar I. Bossier P. Defoirdt T. (2011a). Quorum sensing regulation of virulence gene expression in *Vibrio harveyi in vitro* and *in vivo* during infection of gnotobiotic brine shrimp larvae. Environ. Microbiol. Rep. 3, 597–602. 10.1111/j.1758-2229.2011.00268.x 23761340

[B39] Ruwandeepika H. A. Defoirdt T. Bhowmick P. P. Karunasagar I. Bossier P. (2011b). Expression of virulence genes in luminescent and nonluminescent isogenic vibrios and virulence towards gnotobiotic brine shrimp (*Artemia franciscana*). J. Appl. Microbiol. 110, 399–406. 10.1111/j.1365-2672.2010.04892.x 21091862

[B40] Scarano C. Spanu C. Ziino G. Pedonese F. Dalmasso A. Spanu V.. (2014). Antibiotic resistance of *Vibrio* species isolated from *Sparus aurata* reared in Italian mariculture. New Microbiol. 37, 329–337. Available online at: https://www.ncbi.nlm.nih.gov/pubmed/25180847 25180847

[B41] Shen G. M. Shi C. Y. Fan C. Jia D. Wang S. Q. Xie G. S.. (2017). Isolation, identification and pathogenicity of *Vibrio harveyi*, the causal agent of skin ulcer disease in juvenile hybrid groupers *Epinephelus fuscoguttatus* × *Epinephelus lanceolatus*. J. Fish Dis. 40, 1351–1362. 10.1111/jfd.12609 28252178

[B42] Szpilewska H. Czyz A. Wgrzyn G. (2003). Experimental evidence for the physiological role of bacterial luciferase in the protection of cells against oxidative stress. Curr. Microbiol. 47, 379–382. 10.1007/s00284-002-4024-y 14669913

[B43] Tejedor D. Álvarez-Méndez S. J. López-Soria J. M. Martín V. S. García-Tellado F. (2014). A robust and general protocol for the Lewis-base-catalysed reaction of alcohols and alkyl propiolates. Eur. J. Org. Chem. 2014, 198–205. 10.1002/ejoc.201301303

[B44] Tejedor D. Méndez-Abt G. Cotos L. García-Tellado F. (2013). Propargyl Claisen rearrangement: allene synthesis and beyond. Chem. Soc. Rev. 42, 458–471. 10.1039/C2CS35311C 23034723

[B45] Travers M.-A. Le Bouffant R. Friedman C. S. Buzin F. Cougard B. Huchette S.. (2009). Pathogenic *Vibrio harveyi*, in contrast to non-pathogenic strains, intervenes with the p38 MAPK pathway to avoid an abalone haemocyte immune response. J. Cell. Biochem. 106, 152–160. 10.1002/jcb.21990 19058134

[B46] Trott O. Olson A. (2010). AutoDock VINA: improving the speed and accuracy of docking with a new scoring function, efficient optimization and multithreading. J. Comput. Chem. 31, 455–461. 10.1002/jcc.21334 19499576 PMC3041641

[B47] Visick K. L. Foster J. Doino J. McFall-Ngai M. Ruby E. G. (2000). *Vibrio fischeri lux* genes play an important role in colonization and development of the host light organ. J. Bacteriol. 182, 4578–4586. 10.1128/JB.182.16.4578-4586.2000 10913092 PMC94630

[B48] Waidmann M. S. Bleichrodt F. S. Laslo T. Riedel C. U. (2011). Bacterial luciferase reporters: the Swiss army knife of molecular biology. Bioeng. Bugs 2, 8–16. 10.4161/bbug.2.1.13566 21636983

[B49] Wang L. Chen Y. Huang H. Huang Z. Chen H. Shao Z. (2015). Isolation and identification of *Vibrio campbellii* as a bacterial pathogen for luminous vibriosis of *Litopenaeus vannamei*. Aquac. Res. 46, 395–404. 10.1111/are.12191

[B50] Waters C. M. Bassler B. L. (2006). The *Vibrio harveyi* quorum-sensing system uses shared regulatory components to discriminate between multiple autoinducers. Genes Dev. 20, 2754–2767. 10.1101/gad.1466506 17015436 PMC1578700

[B51] Waters C. M. Lu W. Rabinowitz J. D. Bassler B. L. (2008). Quorum sensing controls biofilm formation in *Vibrio cholerae* through modulation of cyclic di-GMP levels and repression of *vpsT*. J. Bacteriol. 190, 2527–2536. 10.1128/JB.01756-07 18223081 PMC2293178

[B52] Wilder C. N. Diggle S. P. Schuster M. (2011). Cooperation and cheating in *Pseudomonas aeruginosa*: the roles of the *las, rhl* and *pqs* quorum-sensing systems. ISME J. 5, 1332–1343. 10.1038/ismej.2011.13 21368905 PMC3146268

[B53] Xu Y. Wang S. Hu Q. Gao S. Ma X. Zhang W.. (2018). CavityPlus: a web server for protein cavity detection with pharmacophore modelling, allosteric site identification and covalent ligand binding ability prediction. Nucleic Acids Res. 46, W374–W379. 10.1093/nar/gky380 29750256 PMC6031032

[B54] Zhu J. Mekalanos J. J. (2003). Quorum sensing-dependent biofilms enhance colonization in *Vibrio cholerae*. Dev. Cell 5, 647–656. 10.1016/S1534-5807(03)00295-8 14536065

[B55] Zhu Z.-B. Kirsch S. F. (2013). Propargyl vinyl ethers as heteroatom-tethered enyne surrogates: diversity-oriented strategies for heterocycle synthesis. Chem. Commun. (Camb). 49, 2272–2283. 10.1039/c3cc37258h 23340491

