# Supplementary figures and images for: The 9H-Fluoren Vinyl Ether Derivative SAM461 Inhibits Bacterial Luciferase Activity and Protects Artemia franciscana From Luminescent Vibriosis
Source: Front Cell Infect Microbiol. 2018 Nov 8;8:368. doi: 10.3389/fcimb.2018.00368 (PMC6236115; doi:10.3389/fcimb.2018.00368)

**Figure S1**. 1H **(A)** and 13C NMR **(B)** spectra of SAM461 (CDCl3, 400 and 100 MHz, respectively).


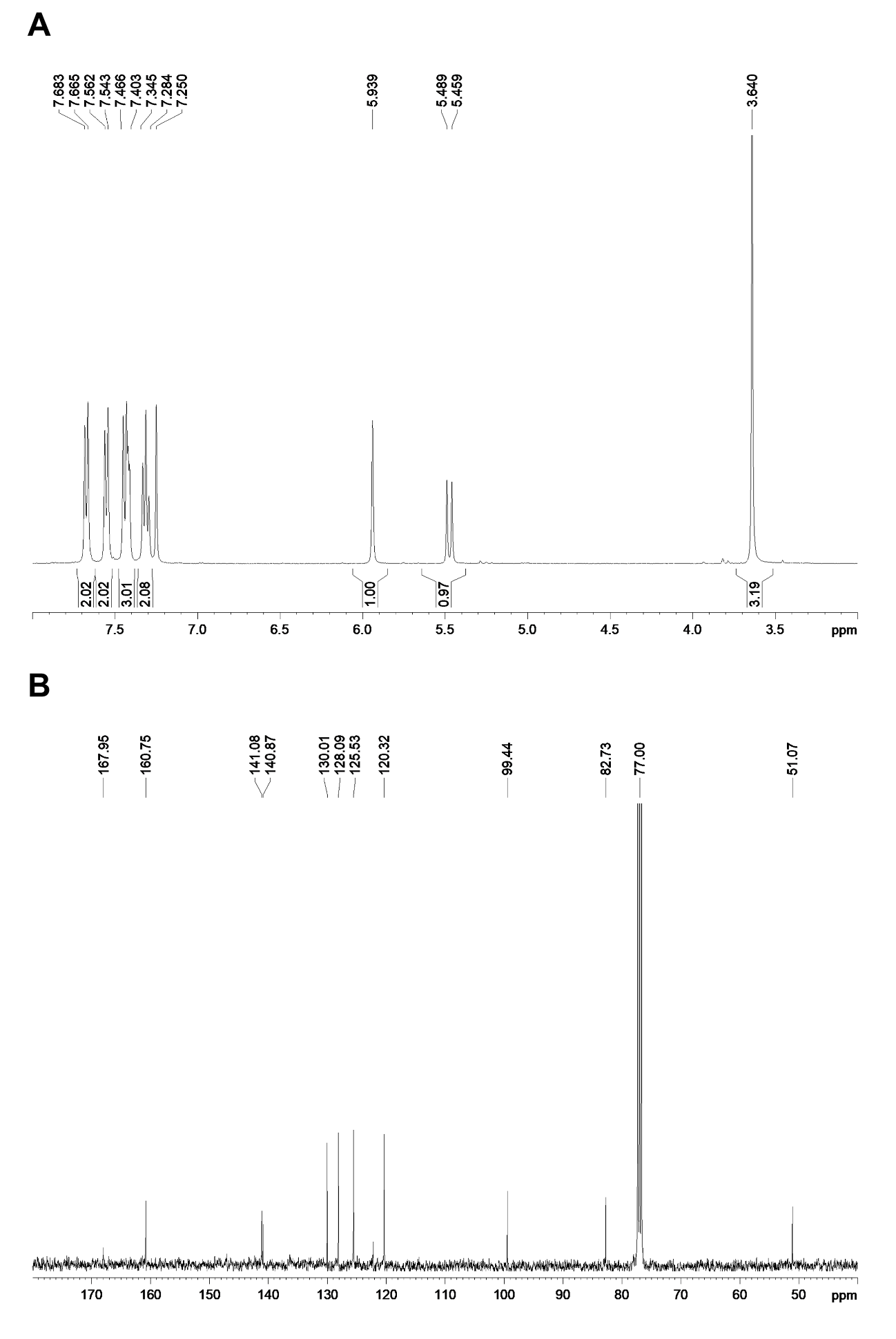

Supplement: Supplementary file 1 [file Data_Sheet_1.docx]
