# Supplementary material for: The 9H-Fluoren Vinyl Ether Derivative SAM461 Inhibits Bacterial Luciferase Activity and Protects Artemia franciscana From Luminescent Vibriosis
Source: Front Cell Infect Microbiol. 2018 Nov 8;8:368. doi: 10.3389/fcimb.2018.00368 (PMC6236115; doi:10.3389/fcimb.2018.00368)

**Figure S2.** **(A)** The X-ray structure of FMNH_2_ (green) compared to the docked pose of FMNH_2_ (purple) in the active site of V_c_Luc (PDB: 3FGC). The highest scoring docking pose of the ligand resembles the X-ray structure. The pyrimidine rings dock similarly, but there is some difference in the flexible aliphatic chain. Interaction diagram of the X-ray structure **(B)** and docked **(C)** FMNH_2_-V_c_Luc complex showing hydrogen bonds and hydrophobic contacts between amino acid residues. **(D)** Interaction diagram of the docked furimazine ligand in the active site of NLuc. This pose offers the ligand only hydrophobic contacts. **(E)** The docked furimazine pose in the potential allosteric site in NLuc. The docking results shows slightly less affinity for this site compared to the active site. **(F)** Docking pose of SAM461 in the potential allosteric site of NLuc. The calculated affinity for the ligand suggests a slight preference towards the active site. Hydrophobic surface areas are colored in magenta, while hydrophilic surfaces are cyan.


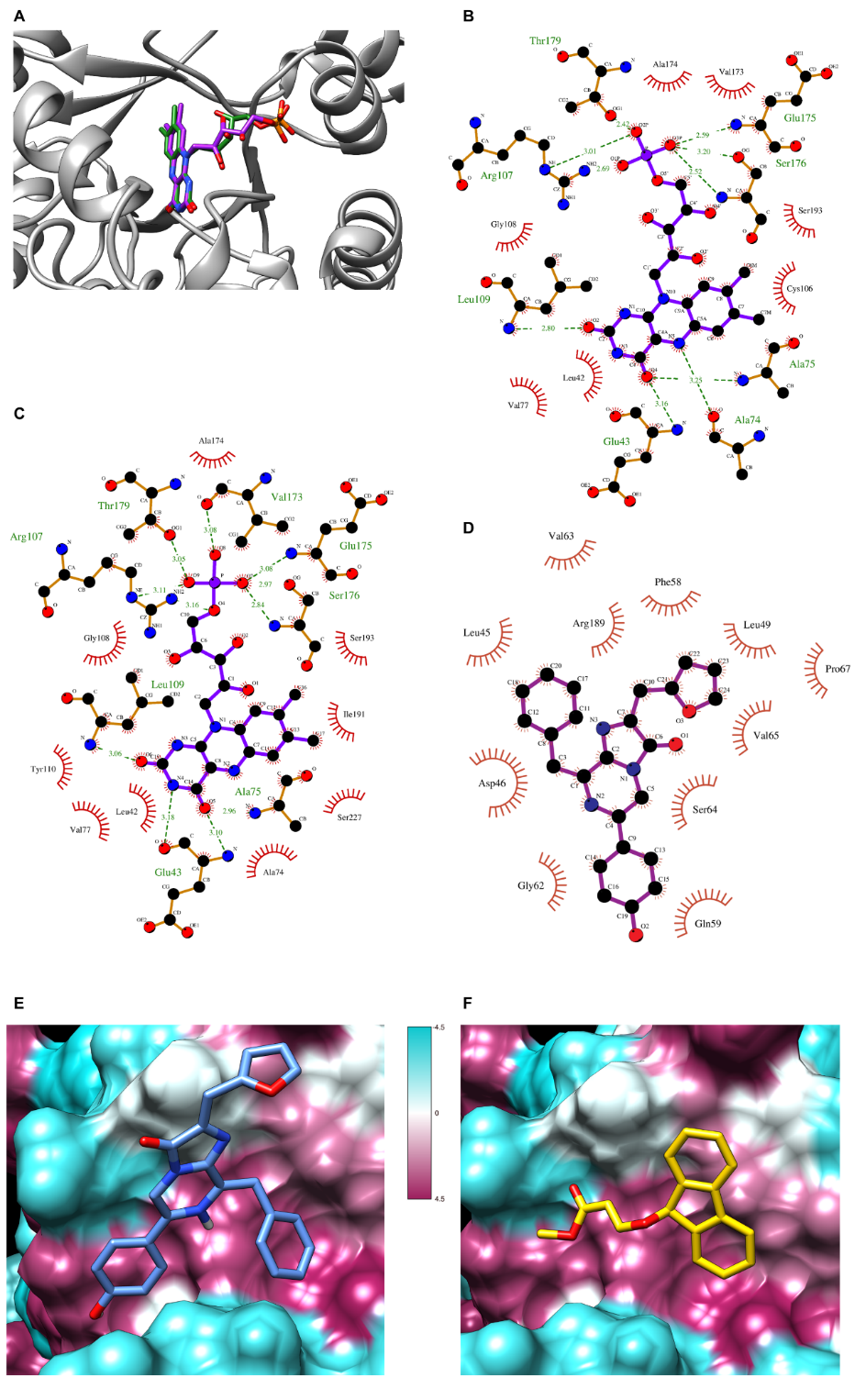

Supplement: Supplementary file 2 [file Data_Sheet_2.docx]
